# Novel Role of Ghrelin Receptor in Gut Dysbiosis and Experimental Colitis in Aging

**DOI:** 10.3390/ijms23042219

**Published:** 2022-02-17

**Authors:** Ji Yeon Noh, Chia-Shan Wu, Jennifer A. A. DeLuca, Sridevi Devaraj, Arul Jayaraman, Robert C. Alaniz, Xiao-Di Tan, Clinton D. Allred, Yuxiang Sun

**Affiliations:** 1 Department of Nutrition, Texas A&M University, College Station, TX 77843, USA; jynoh@tamu.edu (J.Y.N.); chiashan.wu@ag.tamu.edu (C.-S.W.); jdeluca@tamu.edu (J.A.A.D.); cdallred@uncg.edu (C.D.A.); 2 Department of Pathology and Immunology, Baylor College of Medicine, Houston, TX 77030, USA; devaraj@bcm.edu; 3 Artie McFerrin Department of Chemical Engineering, Texas A&M University, College Station, TX 77843, USA; arulj@mail.che.tamu.edu; 4 Department of Microbial Pathogenesis and Immunology, Texas A&M University Health Science Center, College Station, TX 77843, USA; alaniz.tamu08@gmail.com; 5 Department of Pediatrics, Ann & Robert H. Lurie Children’s Hospital of Chicago, Feinberg School of Medicine, Northwestern University, Chicago, IL 60611, USA; xtan@northwestern.edu; 6 Department of Nutrition, University of North Carolina at Greensboro, Greensboro, NC 27412, USA; 7 USDA/ARS Children’s Nutrition Research Center, Department of Pediatrics, Baylor College of Medicine, Houston, TX 77030, USA

**Keywords:** ghrelin, growth hormone secretagogue receptor (GHS-R), aging, microbiome, gut permeability, ulcerative colitis, inflammatory bowel disease (IBD)

## Abstract

Chronic low-grade inflammation is a hallmark of aging, which is now coined as inflamm-aging. Inflamm-aging contributes to many age-associated diseases such as obesity, type 2 diabetes, cardiovascular disease, and inflammatory bowel disease (IBD). We have shown that gut hormone ghrelin, via its receptor growth hormone secretagogue receptor (GHS-R), regulates energy metabolism and inflammation in aging. Emerging evidence suggests that gut microbiome has a critical role in intestinal immunity of the host. To determine whether microbiome is an integral driving force of GHS-R mediated immune-metabolic homeostasis in aging, we assessed the gut microbiome profiles of young and old GHS-R global knockout (KO) mice. While young GHS-R KO mice showed marginal changes in Bacteroidetes and Firmicutes, aged GHS-R KO mice exhibited reduced Bacteroidetes and increased Firmicutes, featuring a disease-susceptible microbiome profile. To further study the role of GHS-R in intestinal inflammation in aging, we induced acute colitis in young and aged GHS-R KO mice using dextran sulfate sodium (DSS). The GHS-R KO mice showed more severe disease activity scores, higher proinflammatory cytokine expression, and decreased expression of tight junction markers. These results suggest that GHS-R plays an important role in microbiome homeostasis and gut inflammation during aging; GHS-R suppression exacerbates intestinal inflammation in aging and increases vulnerability to colitis. Collectively, our finding reveals for the first time that GHS-R is an important regulator of intestinal health in aging; targeting GHS-R may present a novel therapeutic strategy for prevention/treatment of aging leaky gut and inflammatory bowel disease.

## 1. Introduction

Aging is symbolized by chronic low-grade inflammation, thus the term inflamm-aging has been created [1]. Inflamm-aging is linked to many age-associated diseases such as obesity, type 2 diabetes, cardiovascular disease, and various inflammatory diseases. Gut microbiome interacts with the host to modulate intestinal immunity and the host’s disease susceptibility. The microbiota profile is shaped by multifaceted factors, including diet, age, host genetics, environmental factors, and lifestyles; thus, there is huge variation in microbiome signatures among individuals [2,3,4]. Emerging evidence shows that gut microbiome plays an immense role in the biology of aging [5,6]. Nobel Prize Laureate Elie Metchnikoff, the father of immunity, stated that senescence is caused by the poisons originating from human intestinal flora, which underscores the significance of the gut microbiota and the interrelationship between gut microbiome and host aging [7]. We have shown that the intestinal microbiome in aging mice has a unique composition, exhibiting reduced beneficial metabolites, such as tryptophan and indole [8].

Inflammatory bowel disease (IBD) is a chronic inflammatory disease of the intestinal tract. The incidence of IBD has increased over the past 20 years, especially in Western countries and developed Asian countries [9]. There are two types of IBD: ulcerative colitis (UC) and Crohn’s disease (CD) [10]. Clinically, UC and CD share similar symptoms such as abdominal pain, diarrhea, and bloody stool [10]. While the inflammation sites of CD are sporadic throughout the ileum, cecum, and colon, UC primarily involves the colon and rectum [11]. Even though IBD itself is not life threatening [10], it severely affects patients’ quality of life and it is a lifelong disease. Moreover, IBD significantly increases the risk of colorectal cancer in later life [12]. Recent reports revealed that aging increases vulnerability to gastrointestinal disorders [13], and the incidence of IBD in the aging population is on the rise [14,15]. As the age of IBD diagnosis is increasing, IBD patients are more likely to develop proctitis and left-sided colitis [16,17]. Since the etiology of IBD is multifactorial [10,11], currently the therapeutic options are extremely limited. 

Previous studies have revealed that the etiology and pathogenesis of IBD are affected by genetic factors, microbiome composition, and immunological abnormalities [11,18]. Ghrelin is a 28-peptide hormone which is mainly produced by the X/A-like cells in the gastrointestinal tract [19,20]. Ghrelin is known as a hunger hormone; we and others have shown that ghrelin signaling is a major metabolic regulator involved in the pathogenesis of metabolic diseases such as obesity and diabetes [21,22]. However, the effect of ghrelin in intestinal health is controversial; both protective and detrimental effects have been reported [23]. IBD patients, especially UC patients, have high circulating ghrelin [24], and exogenous ghrelin administration has been shown to aggravate experimental colitis [25]. At the same time, other studies showed that ghrelin protects against tissue damage in ulcerative colitis by inhibiting apoptosis of intestinal epithelial cells [26,27]. 

Growth hormone secretagogue receptor (GHS-R), a G-protein receptor, is the biologically relevant receptor for ghrelin. We previously showed that the effects of ghrelin on growth hormone release and food intake are mediated through GHS-R [28], but the role of GHS-R in aging-associated microbiome change and intestinal inflammation is unknown. To decipher the controversial effects of ghrelin signaling on intestinal inflammation and to determine whether GHS-R regulates microbiome-host interaction, in the current study we investigated the role of GHS-R on gut dysbiosis and intestinal inflammation in aging using GHS-R global knockout mice (KO, *Ghsr*^−/−^) with GHS-R ablated in all cell types. We studied the gut microbiome profiles and experimental colitis of both young and aged GHS-R KO mice. 

## 2. Results

### 2.1. Microbiome of Aged GHS-R KO Exhibited Increased Firmicutes and Reduced Bacteroidetes 

**Figure 1:**
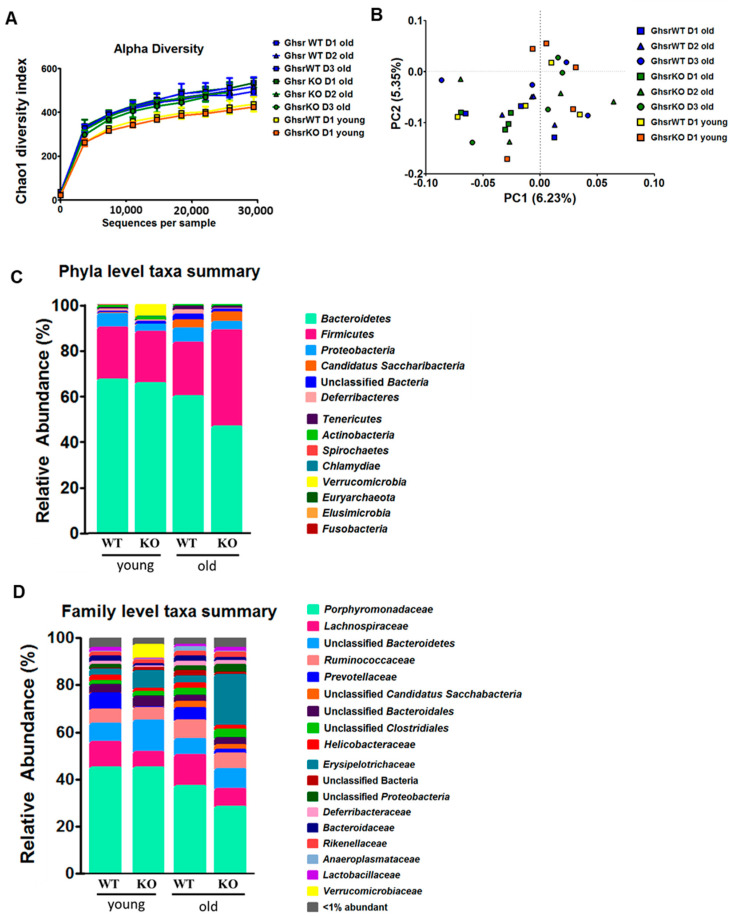
The microbiome profile is shifted in GHS-R KO of both young and aged mice. Reduced Bacteroidetes and increased Firmicutes are more pronounced in aged GHS-R KO rather than young mice. Feces from young (5-month) and old (14-month) male GHS-R KO mice were collected for 3 consecutive days (D1, D2, D3) and analyzed for microbial 16S rRNA. (**A**) α-diversity, (**B**) β-diversity, (**C**) relative abundance (%) at phyla level and (**D**) at family level. *n* = 4–5.

Emerging evidence suggests that the symbiosis between microbiota and their hosts is a new mechanism underpinning the complex host physiology and pathophysiology [29], and that the gut microbiome can be a risk predictor for diseases [30]. To determine whether GHS-R modulates microbiome homeostasis in aging, the feces from young and aged global GHS-R knockout (KO, *Ghsr*^−/−^) and wild-type (WT) were analyzed. To achieve consistency of the data, the feces from aged mice were collected for 3 consecutive days. To investigate how the GHS-R affects the microbiota composition in aging, the α-diversity and β-diversity were analyzed. The Chao1 diversity is the index to assess the number of taxa by predicting the number of low-abundance or missing species [31]. The Chao1 analysis showed higher diversity in aged mice compared to young mice (Figure 1A), which is in line with our previous report [8]. However, a genotype difference was not detected in Chao1 diversity. The β-diversity was also analyzed, whereas no difference was found between age groups or different genotypes (Figure 1B). 

To investigate the effect of GHS-R on microbial colonization in aging, we analyzed microbial 16S rRNA at the phylum and family level (Figure 1C,D). First, we assessed the age effect, by focusing on the common difference between young and old mice (regardless of the genotype). At the phylum level, the dominant phyla were Bacteroidetes, Firmicutes, and Proteobacteria in both young and old mice. The relative abundance of Bacteroidetes decreased with age, whereas the Firmicutes and Proteobacteria maintained similar relative abundance. Moreover, Candidatus Saccharibacteria was more abundant in old compared to young mice (Figure 1C). When the microbiome proportional abundance of feces was analyzed at the family level, Porphyromonadaceae and Prevotellaceae showed a decrease in old mice (Figure 1D).

Second, we assessed the genotype effect by focusing on the difference between the genotypes at young or old age. At the phyla level, Verrucomicrobia was very abundant in young KO mice compared to young WT mice (Figure 1C). At the family level, young GHS-R KO mice showed low Lachnospiraceae and Prevotellaceae, but high Erysipelotrichaceae and Verrucomicrobiaceae (Figure 1D). In the young group, the ratio of Firmicutes and Bacteroidetes (F/B ratio) was similar between young KO and WT mice. Interestingly, Bacteroidetes were lower and Firmicutes were higher in aged KO mice compared to aged WT mice, resulting in increased F/B ratio in the aged KO mice (Figure 1C). Furthermore, aged KO mice had less Porphyromonadaceae, Lachnospiraceae, and Prevotellaceae, but much more Erysipelotrichaceae compared to aged WT mice (Figure 1D). Collectively, these findings suggest that GHS-R regulates microbiome homeostasis, and the effect is more pronounced in aging. 

### 2.2. Ablation of GHS-R Exacerbates DSS-Induced Colitis in Both Young and Aged Mice

Our microbiome data above reveal that the microbiome profile is altered by GHS-R ablation and the effect is exacerbated by aging. A report showed that GHS-R expression in colon is responsive to the treatment of colitis-inducing reagent dextran sulfate sodium (DSS) [32]. Indeed, we have seen GHS-R expression in colon increases in response to DSS in both young and old mice, and aged mice showed more pronounced increase of GHS-R expression (Supplemental Figure S1). This result is in line with the disease-susceptible microbiota profile in aging described above and suggests that GHS-R might be involved in colitis. To test the effect of GHS-R on intestinal inflammation and colitis susceptibility in aging, we induced experimental colitis in both young (4–6-month-old) and aged (18-month-old) male global GHS-R knockout and WT mice. Mice were exposed to 2% (*w*/*v*) DSS for 7 consecutive days. The severity of colitis was evaluated using disease activity index (DAI) score, which includes three criteria: body weight change, rectal bleeding, and fecal consistency, as previously reported [33]. 

#### 2.2.1. DAI of Young Mice

**Figure 2:**
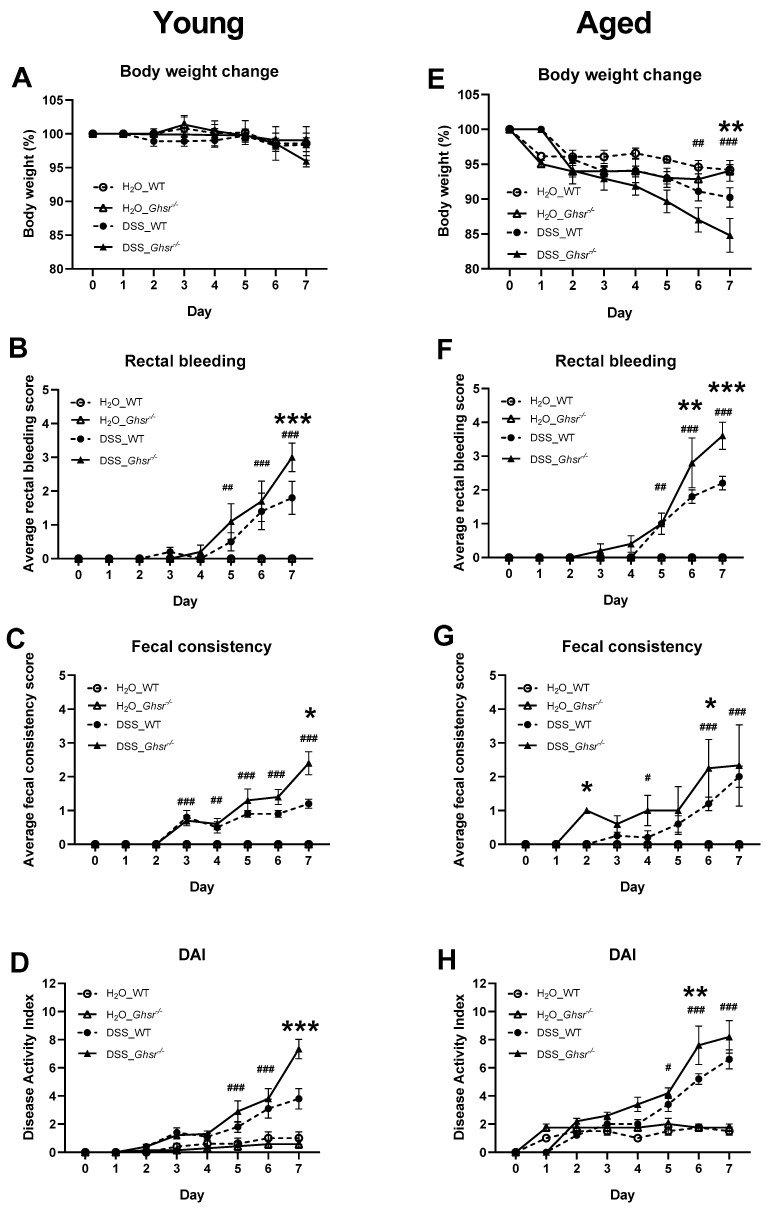
Young and aged GHS-R KO showed exacerbated colitis. Young (4–6-month-old) or old (18-month-old) male mice were exposed to 2% (*w*/*v*) DSS for 7 consecutive days. The left panels are from the young group and the right panels are from the old group. (**A**,**E**) Body weight change (%), (**B**,**F**) rectal bleeding, (**C**,**G**) fecal consistency, (**D**,**H**) disease activity index (DAI) scored by sum of body weight change, fecal consistency, and rectal bleeding. *n* = 5–10 in young group, *n* = 4–5 in aged group. * *p* < 0.05, ** *p* < 0.01, *** *p* < 0.001 WT vs. *Ghsr*^−/−^. #, *p* < 0.05, ##, *p* < 0.01, ### *p* < 0.001 DSS vs. normal water in each genotype.

In young mice, at the start of the study, average body weight of each group was not significantly different (data not shown). Body weight change in young mice was marginal at the beginning of DSS treatment (Figure 2A). However, on day 7, young KO mice showed a noticeable trend of decrease in body weight compared to WT (Figure 2A). 

Young WT mice showed a significant increase of rectal bleeding by 5 days of DSS treatment compared to controls (Figure 2B). Compared to DSS-treated WT, DSS-treated KO mice showed more severe rectal bleeding at the end of the study (Figure 2B). The fecal consistency score of young mice was increased after 3 days of DSS treatment and continued to increase throughout the remaining course of the DSS treatment (Figure 2C). At the end of the study, young KO mice showed a significantly higher fecal consistency score compared to young WT (Figure 2C). Higher rectal bleeding and fecal consistency scores of young GHSR KO mice contributed to the significant increase of DAI scores at the end of DSS treatment (Figure 2D). 

#### 2.2.2. DAI of Old Mice

On the other hand, aged mice showed an average 5% decrease in body weight within 2 days of DSS treatment and continued this reduction throughout the DSS treatment period (Figure 2E). Within 6 days of DSS treatment, body weight was significantly decreased compared to non-DSS treatment in aged mice (Figure 2E). The non-DSS-treated group showed a small reduction of body weight during the study, but did not show a difference between genotypes at the end of the study. The body weight decrease was more pronounced with DSS treatment. The body weight difference between genotypes was evident from day 4 of DSS treatment; at 7 days of DSS treatment, aged KO mice exhibited more significant body weight reduction than their WT counterparts (Figure 2E).

Similar to young WT with DSS treatment, aged WT mice with DSS treatment showed significantly increased rectal bleeding after 5 days of DSS treatment compared to WT controls (Figure 2F). Compared to DSS-treated aged WT, DSS-treated aged KO mice showed significantly more severe rectal bleeding at the end of DSS treatment (Figure 2F). Moreover, aged KO exhibited significantly higher fecal consistency scores after 2 days of DSS treatment and this trend continued until day 6 of DSS treatment (Figure 2G). The greater body weight loss, worse rectal bleeding and fecal consistency scores of aged KO with DSS treatment contributed to the significantly increased DAI (Figure 2H). 

Of note, the rectal bleeding was detected earlier in the aged group than the young group (Figure 2A,E). Moreover, the aged group showed significantly worsened fecal consistency scores earlier in the course of the DSS exposure (Figure 2C,G). At the end of DSS treatment, both young and aged GHS-R KO mice had significantly increased rectal bleeding and worsened fecal consistency scores compared to WT. Unlike young mice, the aged mice given water only showed a slightly increased DAI, less than 2 (Figure 2H), which is likely contributed by the body weight reduction that is likely caused by the acclimatization to the new environment (new cage and new water bottle) as others reported [34]. 

Collectively, a significantly higher DAI was observed in DSS-treated KO mice compared to the WT control in both young and aged groups (Figure 2D,H). The worse DAI score of the KO group was mainly contributed by the increased rectal bleeding and fecal consistency scores, indicating that GHS-R ablation exacerbates DSS-induced colitis. 

#### 2.2.3. Colon Weight/Length and Spleen Weight

**Figure 3:**
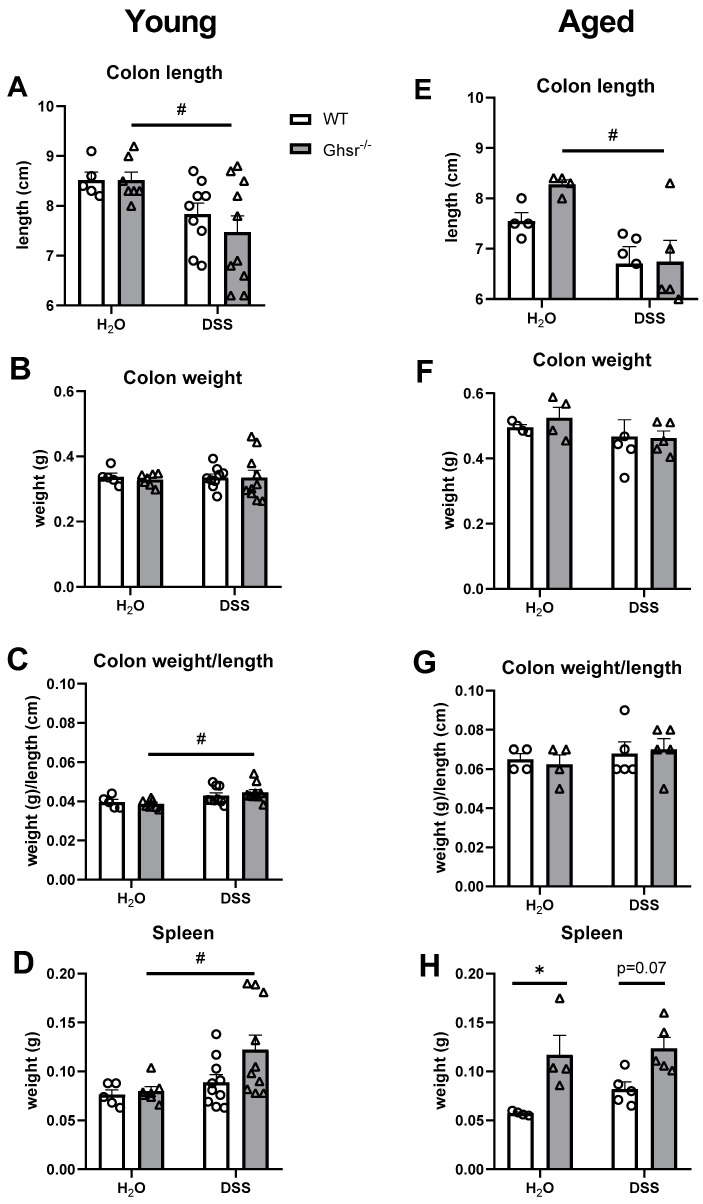
Tissue changes in young and aged *Ghsr^−^*^/*−*^ mice under DSS-induced colitis. Young (4–6-month-old) or aged (18-month-old) male mice were exposed to 2% (*w*/*v*) DSS for 7 consecutive days. The left panels are from the young group and the right panels are from the old group. (**A**,**E**) Colon length, (**B**,**F**) colon weight, (**C**,**G**) colon weight/length ratio on termination day, (**D**,**H**) weight of spleen on termination day. *n* = 5–10 in young group, *n* = 4–5 in aged group. * *p* < 0.05, WT vs. *Ghsr^−^*^/*−*^. # *p* < 0.05, DSS vs. normal water in each genotype.

It is known that colon length is shortened in DSS-induced colitis [34], so we assessed colon weight and length. In the young group, the average colon length of DSS-treated young WT mice had a trend of decrease compared to water-fed WT controls, but it was not statistically significant (Figure 3A). The young KO mice showed a significant decrease in colon length with the DSS treatment (Figure 3A), while colon weight was not affected by DSS treatment compared to water treatment (Figure 3B). The weight/length ratio of colon in young KO mice was significantly increased (Figure 3C), mainly due to the shortened colon length by DSS treatment. However, genotype differences were not seen with either water control or DSS treatment (Figure 3C). 

In the aged group, decreased colon length was observed in DSS treatment (Figure 3E), whereas colon weight was not changed compared to the water control group (Figure 3F). Colon weight/length ratio was not changed by DSS treatment (Figure 3G). Moreover, genotype difference was not found in colon length, weight, or weight/length ratio in both water and DSS groups (Figure 3E–G). Compared to the young group, the aged group had decreased colon length (Figure 3A,E) and increased colon weight (Figure 3B,F). It is noteworthy that while the colon weight and length were not significantly different between two genotypes in either young or old mice, we observed that aging significantly affects the colon length and weight. As shown in Supplemental Figure S2, aging significantly decreased colon length and increased colon weight in aged mice compared to young mice in both water control and DSS-treated groups. Aging increased the colon weight/length ratio compared to young mice mainly due to decreased colon length (Figure 3E,G). 

Besides the colon, we also measured the weight of the spleen, which is one of the key organs involved in inflammation. In the young group, the weight of the spleen was significantly increased in DSS-treated KO compared to control KO; the weight of the spleen of DSS-treated KO had a trend of increase compared to WT, but it did not reach statistical significance (Figure 3D). The spleen weight was slightly higher (not statistically significant) in DSS-treated aged WT mice compared to water controls (Figure 3H). The genotype differences in spleen weights were much more pronounced in the aged group than the young group. Spleen weight was significantly higher in KO mice compared to that of WT in both water control and DSS treatment groups (Figure 3H). 

### 2.3. Ablation of GHS-R Increases Pro-Inflammatory Cytokine Expression and Decreases Gut Tight Junction Proteins in Colon Mucosa

The expression of inflammatory cytokines and gap junction proteins are signatures of intestinal inflammation and intestinal permeability; here, we have assessed pro-inflammatory cytokines and gap junction proteins in young and old GHS-R KO mice under experimental colitis.

**Figure 4:**
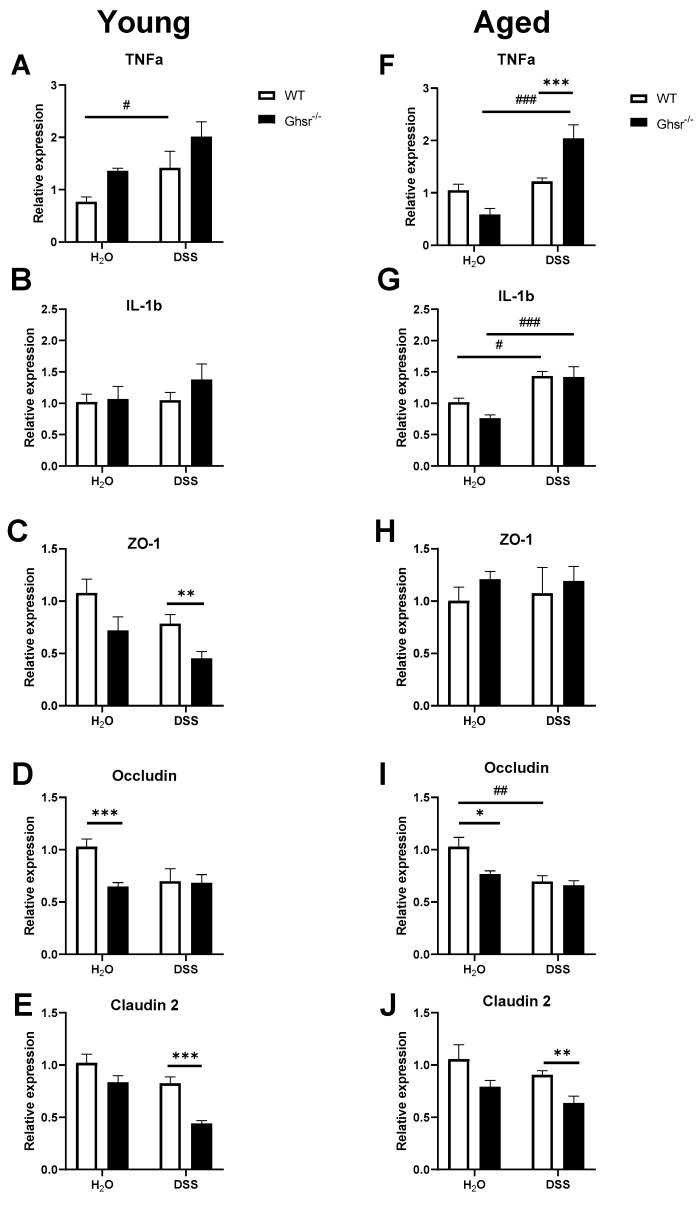
Young and aged *Ghsr^−^*^/*−*^ showed elevated pro-inflammatory cytokines and worsened gut permeability gene expression in colon. Young (4–6-month-old) or aged (18-month-old) male mice were exposed to 2% (*w*/*v*) DSS for 7 consecutive days. The left panels are from the young group and the right panels are from the old group. Expression of proinflammatory cytokines, (**A**,**F**) *TNF*α and (**B**,**G**) *IL-1b* in colon mucosa. Gene expression of tight junction markers: (**C**,**H**) *ZO-1*, (**D**,**I**) *Occludin*, and (**E**,**J**) *Claudin 2*. *n* = 4–5. * *p* < 0.05, ** *p* < 0.01, *** *p* < 0.001, WT vs. *Ghsr^−^*^/*−*^; #, *p* < 0.05, ##, *p* < 0.01, ### *p* < 0.001 DSS vs. control in each genotype.

In the young mice, DSS treatment significantly increased *TNF*α gene expression in the colons of young WT mice (Figure 4A). The young KO group did not show significant differences compared to young WT, but young KO had a trend of higher *TNF*α gene expression compared to WT in both control and DSS groups (Figure 4A). DSS treatment did not alter *IL-1b* expression in the colons of young WT mice (Figure 4B). Moreover, young KO did not show a difference in the *IL-1b* level compared to young WT (Figure 4B). We also tested tight junction markers in the colon to see if alteration of gut permeability contributes to increased inflammation in the colon. DSS treatment showed a trend of decline in expression of *ZO-1*, *Occludin*, and *Claudin-2* (Figure 4C–E). Comparing among genotypes, water control young KO showed a trend of decrease of *ZO-1* compared to WT (Figure 4C); DSS-treated young KO mice showed a significantly decreased *ZO-1* expression compared to WT (Figure 4C). Interestingly, while the expression of *Occludin* was significantly reduced in young KO mice given water, no genotype difference was observed under DSS treatment (Figure 4D). In contrast, young KO given water showed a trend of decrease in *Claudin-2* gene expression compared to WT, but the DSS-treated young KO mice showed significantly decreased *Claudin-2* expression (Figure 4E). 

In the aged group, DSS treatment did not elevate the *TNF*α level in the colons of aged WT mice, but DSS treatment significantly increased the *TNF*α level in aged KO animals compared to mice that received water (Figure 4F). Moreover, *TNF*α gene expression in the aged KO group with DSS treatment was significantly increased compared to aged WT (Figure 4F). In contrast to young mice, DSS treatment significantly increased the level of *IL-1b* in the colons of both aged WT and KO, whereas no genotype difference was detected in either normal water or DSS treatment groups (Figure 4G). DSS treatment did not alter the expression of *ZO-1* (Figure 4H), but it reduced *Occludin* and *Claudin-2* expression (Figure 4I,J) in the aged group. Aged KO mice had significantly decreased *Occludin* in the normal water group compared to aged WT, while a genotype effect was not found with DSS treatment (Figure 4I). DSS-treated aged KO animals also showed significantly decreased *Claudin-2* gene expression compared to WT (Figure 4J). 

Collectively, these data suggest that global ablation of GHS-R elevates colonic inflammation and decreases gut tight junction expression, which is consistent with increased gut permeability and exacerbated colitis.

## 3. Discussion

The gut microbiota coevolve with the host, and the composition of gut microbiota change during aging [8]. Emerging evidence shows that gut microbiota impact the immune responses and affect the onset/susceptibility of many diseases [35,36]. Our finding in the current study reveals that GHS-R plays a critical role in regulating gut microbiota homeostasis and intestinal inflammation in aging. We found that the global ablation of GHS-R promotes gut dysbiosis and increases susceptibility to experimental colitis in aging. 

Our data showed that the aged mice had improved microbial diversity compared to the young mice (Figure 1A), which is in line with previous reports by us and others which showed that the α-diversity is increased in 15- and 19-month-old mice compared to 2-month-old mice [8,37]. Although GHS-R ablation did not affect the microbial community diversity in aging (Figure 1A,B), GHS-R ablation shifted the relative abundance of dominant phyla and families in fecal microbiota towards a proinflammatory profile. At the phylum level, young KO showed increased Verrucomicrobia compared to young WT; at the family level, young KO showed higher abundance of Verrucomicrobiaceae, which is in the family of Verrucomicrobia (Figure 1C). Others reported a high abundance of Verrucomicrobia in biopsy samples from IBD patients [38]. Our study indicates that GHS-R KO mice are more susceptible to experimental colitis, which is consistent with the microbiome profile.

In young mice, GHS-R ablation did not alter the major taxa of Firmicutes and Bacteroidetes, but young GHS-R KO did show decreased Prevotellaceae and increased Erysipelotrichaceae at the family level. The role of Prevotellaceae is still not fully understood [39,40,41], but *Prevotella*, the main genus of the Prevotellaceae family, is thought to be beneficial as its increased abundance is associated with improved glucose metabolism [42]. Erysipelotrichaceae is highly relevant to metabolic disorders including obesity [43,44,45]. Collectively, our findings suggest that the microbial community in young GHS-R KO is prone to an inflammatory state. 

Our data also showed that the aged group had a higher Firmicutes/Bacteroidetes ratio (F/B ratio) as expected (Figure 1C), which is in support of inflamm-aging. Interestingly, aged GHS-R KO mice showed a significantly increased F/B ratio, which is in line with the microbiome profiles observed in diseases such as obesity, hypertension, and stroke [46,47,48]. Higher F/B ratio in the microbiota of aged GHS-R KO suggests that GHS-R ablation modifies the microbiota toward a disease-susceptible state. Indeed, our result showed that aged GHS-R KO mice are more vulnerable to DSS-induced colitis (Figure 4). At the family level, decreased Prevotellaceae and increased Erysipelotrichaceae were observed in aged GHS-R KO mice, similar to that in young GHS-R KO mice (Figure 1D). These data suggest that the effect of GHS-R on microbiome modulation remains throughout the aging process. 

The gut barrier is an interface between the host and microbiome [49]. Consistent with the pro-inflammatory microbiome profile in GHS-R KO mice, we also observed that both young and aged GHS-R KO mice have increased expression of pro-inflammatory cytokines but decreased expression of tight junction markers (Figure 4). Our data are consistent with the reports that age-related dysbiosis of the microbiome exacerbates gut leakiness in aging [5], and experimental colitis is exacerbated in aging [6]. Our data collectively suggest that gut microbiome shifts toward a proinflammatory state in GHS-R KO mice, which likely contributes to the increased gut permeability, primes the gut toward dysbiosis, and increases vulnerability/severity of colitis. 

The functional impact of GHS-R-mediated microbiome changes on susceptibility of colitis could be further determined by cohousing and/or fecal microbiota transplantation (FMT). FMT is an exciting new tool to remodel microbiota that has been used in prevention or treatment of metabolic/inflammatory diseases [50,51,52]. It has been reported that microbiota transplantation from aged mice to young mice increases intestinal permeability and circulating TNF level [5]. Co-housing aged mice with young mice for 10 weeks has been shown to decrease inflammation in liver and spleen of aged mice [53], which may be caused by increased differentiation of pro-inflammatory immune cells in mice receiving FMT from aged mice [54]. Further study of co-housing WT and GHS-R KO and FMT from aged KO to young mice will help to further define the effect of GHS-R mediated microbiome programming on intestinal inflammation and colitis susceptibility.

It also remains to be further determined as to what are the cellular and molecular mechanisms that mediate these GHS-R-induced effects in microbiome regulation and colitis pathogenesis. Intestinal microbiota have been known to produce metabolites such as short chain fatty acids or tryptophan metabolites that are impaired in an age-dependent manner in humans and mice as we and others have reported [8,55,56]. The unique metabolite composition in aging alters immune response, thus exacerbating vulnerability/susceptibility to various age-related diseases [57,58]. Future studies to define how GHS-R affects metabolites would help to elucidate the mechanism of GHS-R mediated microbiome-host interaction in intestinal inflammation and IBD.

Our data showed that aging is associated with decreased Bacteroidetes with similar abundance of Firmicutes, resulting in increased F/B ratio (Figure 1), which is a hallmark microbiome signature of a proinflammatory state. Moreover, compared to young mice, our aged mice had increased Candidatus Saccharibacteria, which is known to change the local microbiome community toward an inflammatory state [59], and is associated with active IBD in humans [60]. To further study whether ablation of GHS-R in aging exacerbates diseases such as IBD, we exposed GHS-R KO and WT mice to 7 days of DSS to induce experimental colitis. We observed that aged mice lost more body weight and showed worse DAI scores sooner during DSS treatment compared to young mice (Figure 2). Similarly, others have reported that old mice have higher F/B ratio in fecal microbiota and more severe DSS-induced colitis [6], and aging exacerbates the severity of colitis in humans and mice [61,62]. Intriguingly, GHS-R KO mice showed worse disease activity index under DSS-induced colitis (Figure 2D,H), increased proinflammatory cytokine expression in the colon (Figure 4A–G), and reduced tight junction gene expression (Figure 4C–J), suggesting that GHS-R-ablated mice are more vulnerable to experimental colitis. We previously showed that aged GHS-R KO mice are lean and insulin sensitive [63,64] and are protected from age-associated impairment of thermogenesis [21]. The unhealthy microbiome profile and severe colitis phenotype observed in aged GHS-R KO mice in the current study are contrary to the healthy metabolic phenotype previously reported under normal aging, which suggests that the effect of GHS-R in colon is different from that in adipose tissue and its effect in the intestinal system is likely dependent on the health state of the intestine. 

The study of GHS-R in the intestinal system is very limited. Only one report showed attenuated experimental colitis in GHS-R ablated mice [32]. This result is different from ours in that both young and old GHS-R KO mice displayed more severe DSS-induced colitis than controls. Our GHS-R KO mice have been backcrossed onto a pure C57BL/6J background and we studied them under both young (4–6 months) and old (18 months) ages. The report by others used GHS-R ablated mice with mixed backgrounds of 129S3/SvImJ and C57BL/6J at the very young age of 2 months old only [32]. That has shown that the susceptibility to DSS-induced colitis in mice is different depending on the genetic background [65,66,67]. It has been reported that C57BL/6 mice have greater body weight decrease and more severe diarrhea and fecal bleeding under DSS treatment than BALB/c mice [67]. However, it is not known if susceptibility to DSS-induced colitis differs between mice of C57BL/6J and 129S3/SvImJ backgrounds. Moreover, the age of the mice may affect the development of colitis. UC could develop at any age, but the peak incidence of colitis is 20–30 years old in humans [68]. Therefore, in the current study, we used 4–6-month-old mice as the young group which is equivalent to 20–30 years old in humans. More studies are needed to further investigate the role and mechanism of GHS-R on intestinal inflammation in different ages and under different genetic backgrounds. 

Lastly, given that the mouse model used in the current study is a global ablation of GHS-R, several types of cells that express GHS-R may contribute to the outcome of gut microbiome and DSS-induced colitis. We and others previously reported that the highest level of GHS-R expression was detected in the brain, with low expression in several peripheral tissues [69,70], suggesting the possibility of involvement of the GHS-R mediated gut-brain axis in the pathogenesis of DSS-induced colitis. We previously showed that GHS-R is also expressed in peritoneal macrophages, approximately 60% of the highest expression tissue hypothalamus [71]. In addition to macrophages, other immune cells such as monocytes, dendritic cells, neutrophils, natural killer cells, and T and B lymphocytes also express GHS-R [72,73,74,75], which may all contribute to the phenotype we observed in the global KO mice. Of note, the colon is where the most immune cells reside in the body and age-related downregulation of host inflammatory immune responses in the colon has been suggested to be a major contributor to disease pathogenesis and progression [37]. In our current mouse model, we were not able to differentiate the effect of GHS-R in specific cell types. Cell-specific deletion of GHS-R in immune cells would be of great interest for further mechanistic understanding. 

## 4. Materials and Methods

### 4.1. Animal Model 

We previously reported the generation of GHS-R KO mice (*Ghsr*^−/−^) that has been fully backcrossed onto the C57BL/6J background [28,63]. The mice were housed and bred at the laboratory animal facility at Texas A&M University under controlled lights and temperature (12 h light-dark cycle, 75 ± 1 °F) with free access to regular diet—Harlan-Teklad 2018X (Harlan-Teklad, Madison, WI, USA) that contains 18% of calories from fat, 58% from carbohydrates, and 24% from protein. To determine the age-associated gut dysbiosis, young (4–6-month-old, *n* = 5–10) and aged (18-month-old, *n* = 4–5) male mice were studied. The mice were randomly divided into two groups and given either water (H_2_O) or 2% (*w*/*v*) dextran sulfate sodium (DSS) solution.

### 4.2. DSS-Induced Colitis

To induce experimental colitis, 2% (*w*/*v*) DSS (MP Biomedicals; 36–50 kDa, Santa Ana, CA, USA) was provided in drinking water for 7 days ad libitum, and DSS was replaced every 48 h to maintain freshness and potency. Body weight loss, fecal consistency, and rectal bleeding were scored daily based on the scoring system reported by co-author Dr. Allred’s group [33]. Briefly, body weight change was scored as 0: body weight gain or 0–1% loss, 1: 1–5% loss, 2: 5–10% loss, 3: 10–15% loss, 4: >15% loss compared to day 0 of the study. Fecal consistency was scored as 0: normal stool, 1: soft but formed pellet, 2: very soft pellet, 3: diarrhea (no pellet), or 4: dysenteric diarrhea (blood in diarrhea). Rectal bleeding was scored as 0: no bleeding, 2: presence of visible blood in stool (red/dark pellet), 4: gross macroscopic bleeding (blood around anus). 

### 4.3. Feces Collection and 16S rRNA Microbiota Analysis 

Animals were single housed for 4 days and the feces were collected for 3 consecutive mornings. Feces were flash frozen then stored at −80 °C until analysis was performed. The microbiome analysis was conducted as we described previously [76]. In brief, nucleic acid extraction of feces was performed using MO BIO PowerSoil extraction kit (MO BIO Laboratories, Carlsbad, CA, USA). NEXTflex 16S V4 ampliconSeq Kit 2.0 (Bio Scientific, Austin, TX, USA) was used to sequence the V4 region of the 16S rRNA gene. The sequences of the 16S V4 forward and reverse primes are: 16S V4 forward: GACGCTCTTCCGATCTTATGGTAATTGTGTGCCAGCMGCCGCGGTAA; reverse: TGTGCTCTTCCGATCTAGTCAGTCAGCCGGACTA CHVGGGTWTCTAAT.

A minimum of 800 and an average of 7500 sequences per sample were generated on Illumina MiSeq platform (Illumina, San Diego, CA, USA). The processing of sequencing data was performed as previously reported [77]. Sequence data were processed through the LotuS pipeline, and the UPARSE algorithm was used to decrease error rates as a quality filter. For taxonomic assignment, RDP was used as the classifier, and HItDB and SILVA were utilized as the selected databases. Chao1 alpha diversity was generated using QIIME 1.7. Beta diversity was calculated by the unweighted UniFrac distance and Bray–Curtis dissimilarity. Phylum and family taxonomic levels were compared between the groups.

### 4.4. Tissue Collection, RNA Extraction, and Quantitative Real-Time PCR 

Colons were collected and washed with ice-cold saline and then opened longitudinally. The proximal 1 cm in colon was scraped and homogenized in RNA lysis solution from Aurum™ Total RNA mini kit (Bio-Rad Laboratories, Inc., Hercules, CA, USA). Samples were stored at −80 °C until further analysis. Total RNA was isolated using Aurum™ Total RNA mini kit (Bio-Rad Laboratories, Inc., Hercules, CA, USA) following the manufacturer’s instructions. For reverse transcription, iScript™ Reverse Transcription Supermix (Bio-Rad Laboratories, Inc., Hercules, CA, USA) was used to synthesize complementary DNA according to the manufacturer’s instructions. Quantitative real-time PCR was performed using SsoAdvanced™ Universal SYBR (Bio-Rad Laboratories, Inc., Hercules, CA, USA) and CFX384 Touch™ Real-Time PCR Detection System (Bio-Rad Laboratories, Inc., Hercules, CA, USA). The primer sequences for GHS-R are: GHS-R-1a forward: 5′-GGACCAGAACCACAAACAGACA-3′; GHS-R-1a reverse: 5′-CAGCAGAGGATGAAAGCAAACA-3′. The rest of the primer information is available upon request. 

### 4.5. Statistical Analysis 

Data were analyzed using GraphPad Prism 8.0.1 (GraphPad Software, La Jolla, CA, USA) and presented as mean ± SEM. Student’s *t*-test, two-tailed t-test, one-way or two-way analysis of variance (ANOVA) with Tukey’s post hoc test were used. * *p* < 0.05 was considered statistically significant; ** *p* < 0.01; *** *p* < 0.001. 

## 5. Conclusions

Here, we investigated the novel effects of GHS-R on microbiome homeostasis and experimental colitis in aging. Both young and old global GHS-R KO mice were more vulnerable to experimental colitis, showing increased proinflammatory cytokines and reduced gut tight junction expressions. We observed that GHS-R suppression shifted the gut microbiota towards a proinflammatory state, which likely contributes to increased susceptibility to experimental colitis. Our findings highlight the relevance of GHS-R in the modulation of the microbial community in aging and underscore the importance of GHS-R in intestinal health in aging. Targeting GHS-R may present a novel therapeutic strategy for prevention and treatment of aging leaky gut and inflammatory bowel disease.

## Data Availability

Data are available upon request from the corresponding author.

## Supplementary Materials

The following supporting information can be downloaded at: https://www.mdpi.com/article/10.3390/ijms23042219/s1.
