# Supplementary material for: Novel Role of Ghrelin Receptor in Gut Dysbiosis and Experimental Colitis in Aging
Source: Int J Mol Sci. 2022 Feb 17;23(4):2219. doi: 10.3390/ijms23042219 (PMC8875592; doi:10.3390/ijms23042219)
Supplement: Supplementary file 1 [file ijms-23-02219-s001.zip › ijms-1594946-supplementary.pptx]

## Slide 1
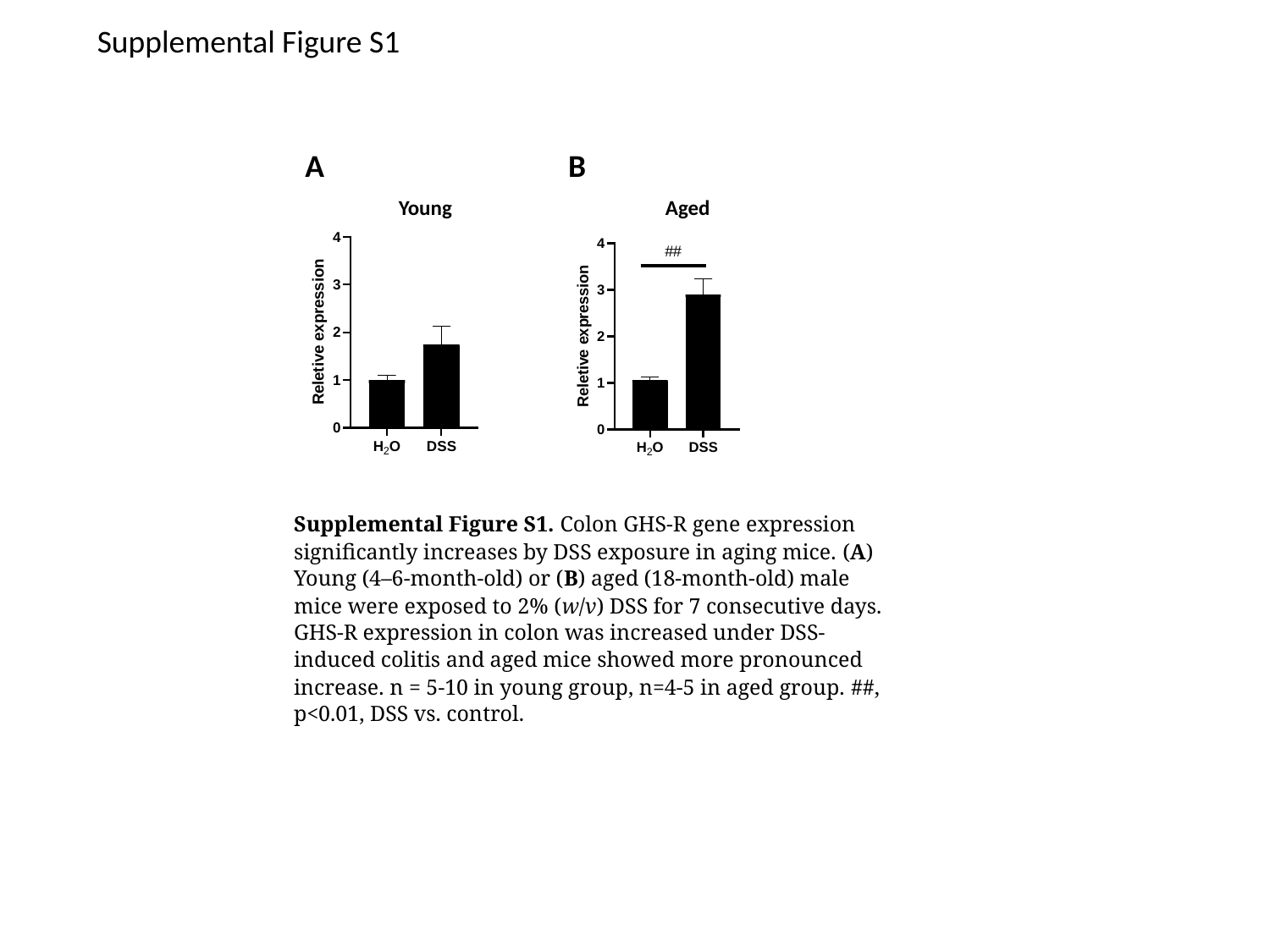

Supplemental Figure S1
A B
 Young Aged
Supplemental Figure S1. Colon GHS-R gene expression significantly increases by DSS exposure in aging mice. (A) Young (4–6-month-old) or (B) aged (18-month-old) male mice were exposed to 2% (w/v) DSS for 7 consecutive days. GHS-R expression in colon was increased under DSS-induced colitis and aged mice showed more pronounced increase. n = 5-10 in young group, n=4-5 in aged group. ##, p<0.01, DSS vs. control.

## Slide 2
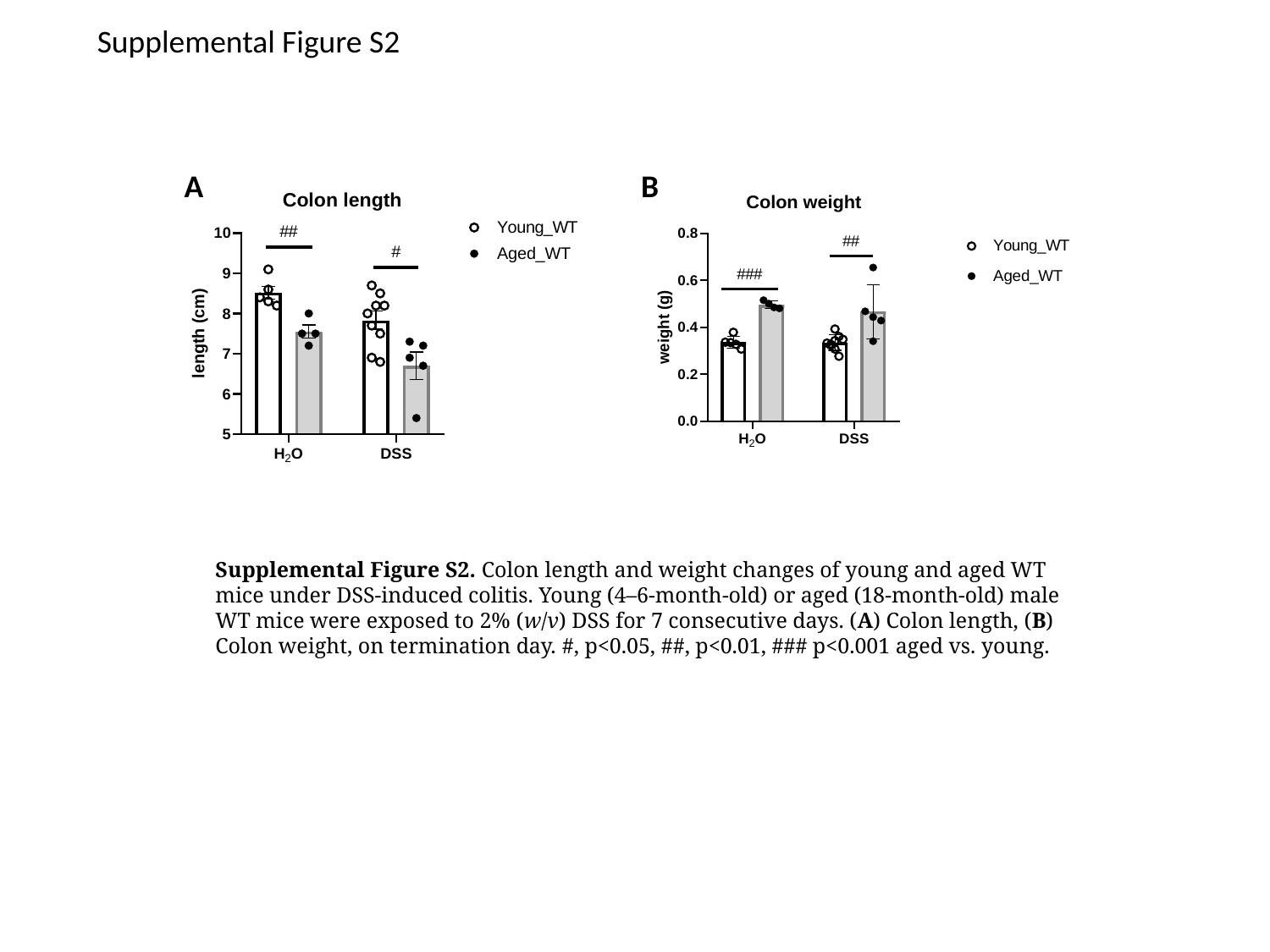

Supplemental Figure S2
A B
Supplemental Figure S2. Colon length and weight changes of young and aged WT mice under DSS-induced colitis. Young (4–6-month-old) or aged (18-month-old) male WT mice were exposed to 2% (w/v) DSS for 7 consecutive days. (A) Colon length, (B) Colon weight, on termination day. #, p<0.05, ##, p<0.01, ### p<0.001 aged vs. young.
